# Food for Bone: Evidence for a Role for Delta-Tocotrienol in the Physiological Control of Osteoblast Migration

**DOI:** 10.3390/ijms21134661

**Published:** 2020-06-30

**Authors:** Lavinia Casati, Francesca Pagani, Roberto Maggi, Francesco Ferrucci, Valeria Sibilia

**Affiliations:** 1 Department of Medical Biotechnology and Translational Medicine, Università degli Studi di Milano, 20129 Milano, Italy; lavinia.casati@unimi.it (L.C.); francesca.pagani@unimi.it (F.P.); 2 Department of Pharmaceutical Sciences, Università degli Studi di Milano, 20133 Milano, Italy; roberto.maggi@unimi.it; 3 Department of Health, Animal Science and Food Safety, Università degli Studi di Milano, 20133 Milano, Italy; francesco.ferrucci@unimi.it

**Keywords:** delta-tocotrienol, MC3T3-E1 cells, osteoblast migration, Wnt/β-catenin pathway, H3 and H4 acetylation level

## Abstract

Bone remodeling and repair require osteogenic cells to reach the sites that need to be rebuilt, indicating that stimulation of osteoblast migration could be a promising osteoanabolic strategy. We showed that purified δ-tocotrienol (δ-TT, 10 μg/mL), isolated from commercial palm oil (*Elaeis guineensis*) fraction, stimulates the migration of both MC3T3-E1 osteoblast-like cells and primary human bone marrow mesenchymal stem cells (BMSC) as detected by wound healing assay or Boyden chamber assay respectively. The ability of δ-TT to promote MC3T3-E1 cells migration is dependent on Akt phosphorylation detected by Western blotting and involves Wnt/β-catenin signalling pathway activation. In fact, δ-TT increased β-catenin transcriptional activity, measured using a Nano luciferase assay and pretreatment with procaine (2 µM), an inhibitor of the Wnt/β-catenin signalling pathway, reducing the wound healing activity of δ-TT on MC3T3-E1 cells. Moreover, δ-TT treatment increased the expression of β-catenin specific target genes, such as Osteocalcin and Bone Morphogenetic Protein-2, involved in osteoblast differentiation and migration, and increased alkaline phosphatase and collagen content, osteoblast differentiation markers. The ability of δ-TT to enhance the recruitment of BMSC, and to promote MC3T3-E1 differentiation and migratory behavior, indicates that δ-TT could be considered a promising natural anabolic compound.

## 1. Introduction

Today, lifestyle represents the most natural approach to fight the increasing susceptibility to several diseases, including osteoporosis. Osteoporosis is a skeletal disorder characterized by low bone mass and microarchitectural deterioration of bone tissue, leading to enhanced bone fragility and a consequent increase in fracture risk [1].

Bone structural integrity is maintained by the removal of old bone by osteoclasts and the synthesis of new bone in its place by osteoblasts. The assembly of osteoblasts and osteoclasts into temporary anatomic structures called basic multicellular units (BMU) accomplishes this process called remodeling. After age 40, negative BMU balance, due to a persistent excess of bone resorption over the formation, marks the onset of age-related bone loss.

The identification of new strategies able to stimulate bone formation represents one of the more exciting goals of basic research in the bone field. In the clinic, the use of anabolic agents could be useful not only to restore the bone mass in the course of osteoporosis but also to improve bone repair and bone regeneration.

Accumulating evidence indicates that osteoblast migration exerts a pivotal role in the physiological control of bone metabolism, including responses to mechanical loading [2]. 

Besides its fundamental role in skeletal physiology, the osteoblast migration is involved in pathological conditions, including tumor metastasis and repair of bone fractures [3].

During fracture repair, osteogenic cells that derive either from circulation or resident stem cell pools are required to migrate along osseous surfaces and navigate to reach their destination, i.e., the site of new bone formation. Thus, a failure of osteoblasts to infiltrate and populate the callus tissue formed after an injury may contribute to impaired fracture repair.

Even if the migratory potential of osteoblastic cells was described in 1977 by Jones and Boyde [4], relatively little remains known about the identification of new compounds able to increase bone formation by improving osteoblast migration. This is surprising since current anabolic therapies are aimed to increase osteoblast maturation and differentiation.

Recently, a great deal of interest has been focused on active plant-derived products to improve or maintain bone health. These natural compounds have been used not only in experimental in vivo and in vitro studies but also in clinical practice since they have fewer adverse reactions and are more suitable for long-term treatments than chemically synthesized compounds [5,6].

Among natural compounds which received much attention there are the compounds derived from vitamin E like tocotrienols (TTs). There are four distinct isomers (α, β, γ, and δ) in TTs depending on the position of the methyl group on the chromanol ring.

Regarding the potential benefits of TTs on bone tissue, TTs mixture [7], purified γ-TT [8], or δ-TT [9] have been reported to protect against experimental models of osteopenia [10,11]. More interestingly, osteoporosis has been correlated with low intake, and serum levels of TTs [12] and clinical studies have reported that dietary supplementation of TTs extracted from annatto (*Bixa Orellana*) seeds (consisting of 90% δ-TT and 10% γ-TT) exerts a beneficial action on bone turnover in postmenopausal women [13]. Annatto TTs exert an inhibitory action on bone resorption and osteoclast differentiation [8]. Furthermore, annatto TTs have been shown to enhance the expression of genes involved in both bone formation and osteoblast activity [14,15]. In our previous studies, we have shown that purified δ-TT exerts a stimulatory effect on MC3T3-E1 cell viability and protects MC3T3-E1 cells against oxidative stress [16] suggesting that δ-TT could represent a valuable approach in the prevention and management of osteoblast dysfunction.

However, the possible effect of δ-TT on osteoblast motility remains to be clarified. The present study was aimed to address the effects of δ-TT on osteoblast-like MC3T3-E1 cells migration and the mechanisms involved in the stimulatory action of δ-TT on MC3T3-E1cell migration. We first examined the role of PI3K/akt pathway which plays a role in the actin cytoskeleton reorganization and cell migration. We then studied the Wnt/β-catenin pathway, which promotes the expression of genes involved not only in osteoblast differentiation and proliferation [17], but also in cellular motility [18]. Among them, we examined the influence of δ-TT on the expression of OC, collagen I and BMP-2 and its ability to stimulate MC3T3-E1 cell differentiation. We performed additional experiments to study the ability of δ-TT to promote the motility of human bone marrow mesenchymal stem cells (BMSCs), multi potential progenitor cells which are recruited to the bone surface during bone repair and then differentiate into osteogenic cells.

Finally, we examined the effect of δ-TT on the level of global acetylation of histone H4 and H3, an important epigenetic mark of gene activation, since rrecent evidence indicates that the bone epigenome exerts a fundamental role in the control of bone cell activities [19]. 

## 2. Results

### 2.1. Effects of δ-TT on MC3T3-E1 Cell Cycle

**Figure 1:**
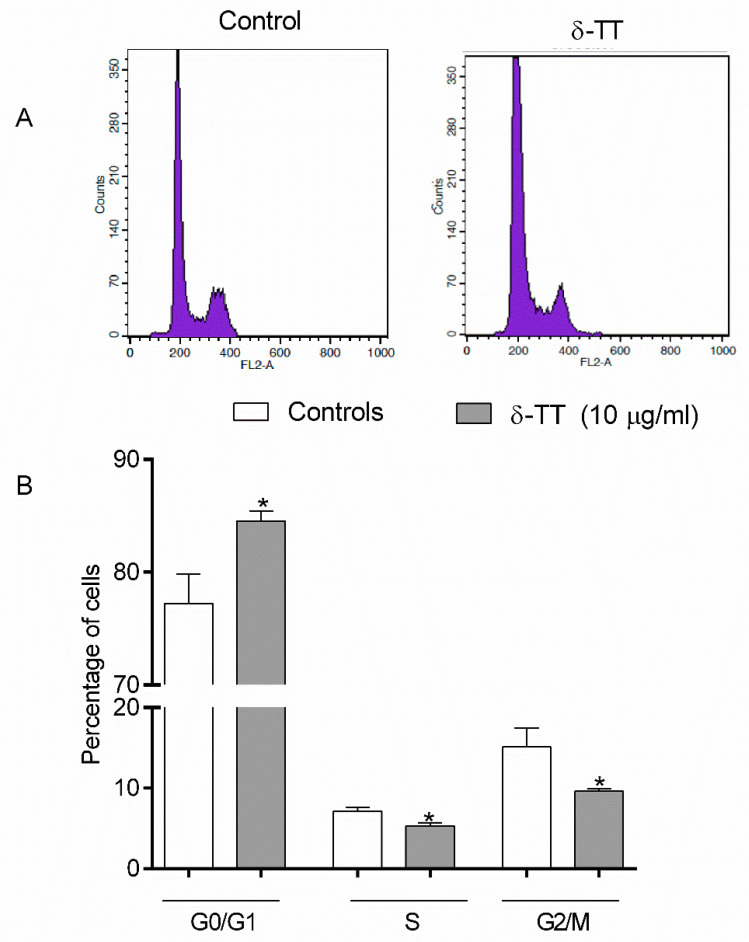
Effects of δ-TT (10 µg/mL) on the different cell cycle phases in MC3T3-E1 cells after 24 h of incubation. The values are the percentage of cells in each phase, as determined by the FACScalibur flow cytometer. (**A**) representative FACS analysis images; (**B**) Quantification of cell cycle distribution. The bars represent the mean ± SEM of six replicates within a single experiment. * *p* < 0.05 vs. controls.

We have investigated the effects of 24 h treatment with δ-TT on cell cycle phases in MC3T3E1. The percentage of cells in each cell cycle phase (G_0_/G_1_, S, and G_2_/M) was determined by flow cytometry and PI DNA staining. We used δ-TT at the concentration (10 μg/mL) previously shown to increase MC3T3-E1 cell viability [16]. As shown in Figure 1, δ-TT significantly increased the percentage of cells in G_0/_G_1_ and reduced the percentage of the cell in both S and n G_2/_M phases.

### 2.2. Effect of δ-TT on MC3T3-E1 and BMSC Cell Migration

**Figure 2:**
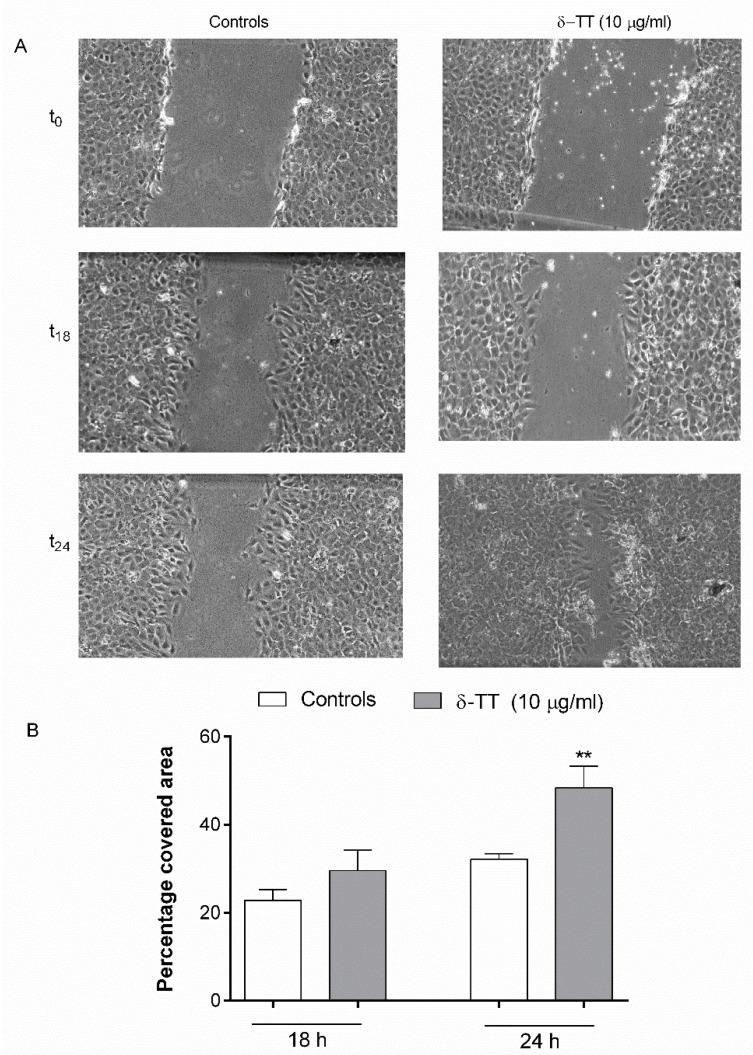
δ-TT (10 µg/mL) promotes the migration of MC3T3-E1 cells in wound healing assay. δ-TT treatment was performed 24 h before and immediately after cells were scratched. (**A**) Wound healing assay of untreated and treated MC3T3-E1 cells; (**B**) Quantification of wound closure. The graph represents the wound width as the % of the closure of the original wound. Wound size was detected using ImageJ Software. The data are presented as the mean ± SEM of three independent experiments. ** *p* < 0.01 vs. controls.

We then studied the effects of δ-TT on cell migration, performing a scratch assay. MC3T3-E1 cells were exposed to δ-TT 24 h before and 24 h after the wound gap has been applied. As shown in Figure 2, MC3T3-E1 cells treated with δTT exhibited a faster wound healing rate compared to that of control untreated cells at 18 and 24 h, reaching a statistical significance at 24 h.

**Figure 3:**
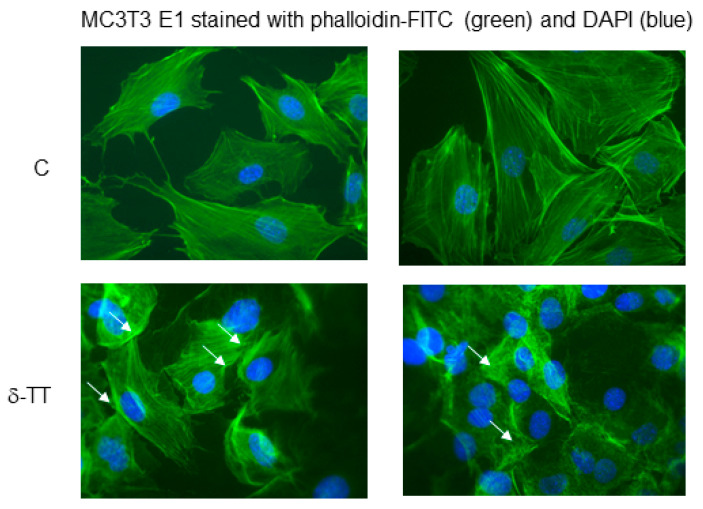
δ-TT (10 µg/mL) induces cytoskeleton remodeling in MC3T3E1. We have performed the staining for F-actin (using FITC phalloidin) to visualize the cytoskeleton remodeling (white arrow). The nuclei were stained with DAPI. Magnification 40× in a Zeiss Axiovert microscope equipped for fluorescence analysis with the corresponding sets of filters to detect FITC and DAPI emission.

When we examined the effects of δ-TT in the organization of the actin cytoskeleton mediating cell migration, we found that cells treated with δ-TT presented cortical protrusions enriched in actin, in comparison with control-treated cells, which are closely related to cell locomotion (Figure 3). 

**Figure 4:**
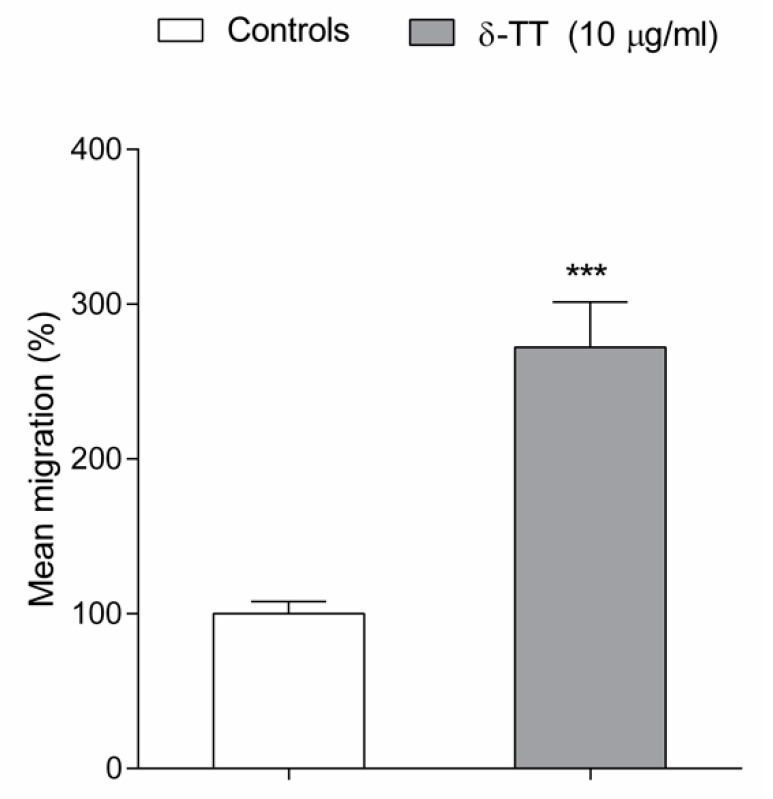
δ-TT (10 µg/mL) promotes the BMSC cell migration in a Boyden chamber assay. BMCS were exposed to δ-TT 24 h before and during the micro-chemotaxis assay. The migrated cells were counted by selecting three random fields at 10× or 20× objective on an optical microscope. Data are reported as mean percentage of migrated δ-TT exposed cells versus control cells. *** *p* < 0.001 vs. controls.

Lastly, we examined the effects of δ-TT on BMSC cell migration, performing a micro-chemotaxis assay in a Boyden chamber. BMCS exposed to δ-TT 24 h before and during micro-chemotaxis assay, showed a significant increase in cell migration (170%) in comparison to the control-treated cells (Figure 4).

### 2.3. Involvement of Akt Pathway in δ-TT-Induced MC3T3-E1 Cell Migration

To study the molecular pathways involved in the effects of δ-TT on MC3T3-E1 cells, we first examined the PI3K/Akt pathway, which regulates actin reorganization during cell migration. The ability of δ-TT to activate PI3K was studied by examining a) the phosphorylation status of Akt, which correlates with Akt activation by PI3K and b) the effects of δ-TT on cell migration in MC3T3-E1 cells pretreated with LY294002, a PI3K/Akt specific inhibitor.

**Figure 5:**
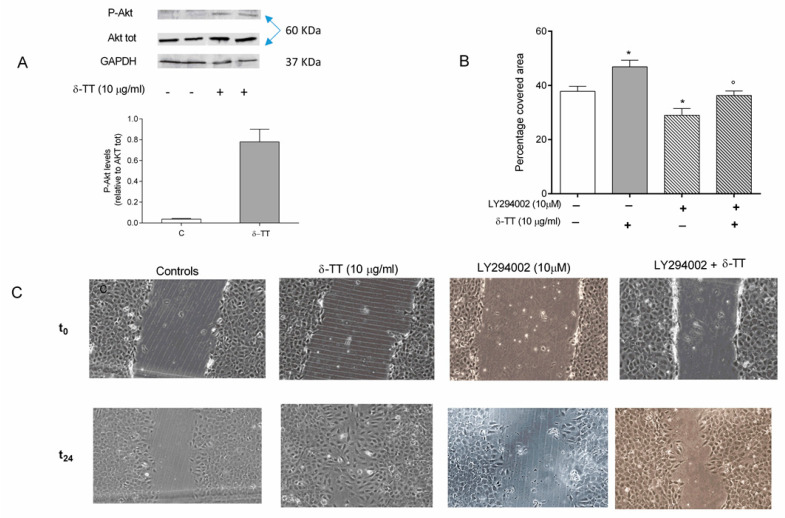
Involvement of PI3-K/AKT pathway in the wound healing effect of δ-TT (10 µg/mL) on MC3T3-E1 cells. (**A**) Cells were pretreated with δ-TT for 24 h, proteins were collected and levels of Akt phosphorylation (Ser473) and of total Akt were analysed by Western Blotting. Densitometer analysis of the bands was performed with ImageLab 4.0 provided by Biorad. We have reported a ratio between Phospho Akt/Akt total densitometer analysis. (**B**,**C**) pretreatment with LY294002 (10µM), a PI3-K antagonist, removes the wound healing effects of δ-TT. Cells were incubated with LY294002 1 h min before treatment with δ-TT. The Wound healing assay was performed as detailed in Figure 2. The data are presented as the mean ± SEM of three independent experiments. * *p* < 0.05 vs. controls; ° *p* < 0.05 vs. δ-TT.

As shown in Figure 5A, δ-TT induces Akt phosphorylation, detected by Western blotting, in MC3T3-E1 cells, indicating the involvement of the PI3/Akt pathway in the δ-TT activities.

LY294002 (10 µM, 1h before δ-TT) significantly worsened the migratory capacity of MC3T3-E1 cells and removed the wound healing action of δ-TT (Figure 5B,C).

### 2.4. Beta-Catenin Is Involved in the Effect of δ-TT on Cell Migration

We have investigated the effect of δ-TT on β-catenin transcriptional activity in MC3T3-E1 cells by using a gene reporter assay. We used two different plasmids: one containing a Nano luciferase gene under the control of the TCF/LEF responsive element, sensitive to the β-catenin activation, the other construct is codifying for luciferase gene, and it is under the control of a strong promoter. Both plasmids are commercially available from Promega.

**Figure 6:**
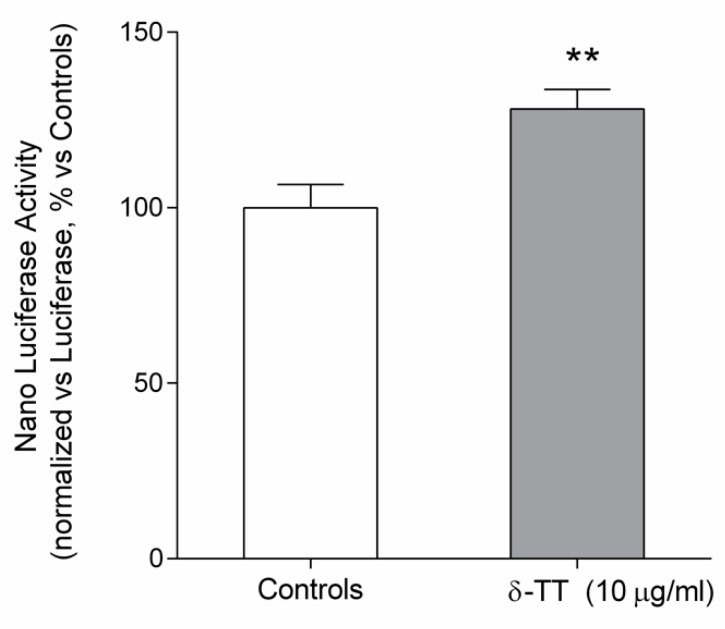
Involvement of β-catenin transcriptional activity in the effect of δ-TT in MC3T3E1 cells transiently cotransfected with pGL4.54 (luc2TK) pNL (NLucP/TCF-LEF-RE) and treated for 48 h with δ-TT (10 µg/mL). The ratio between Nano luciferase activity and luciferase activity (luminescence, arbitrary units) was expressed as percentage vs. controls. The data are presented as the mean ± SEM of eight replicates. ** *p* < 0.01 vs. controls.

As reported in Figure 6, δ-TT induced β-catenin transcriptional activity, reflected by a statistical increase in the luciferase activity as compared to that of control-untreated MC3T3-E1 cells.

**Figure 7:**
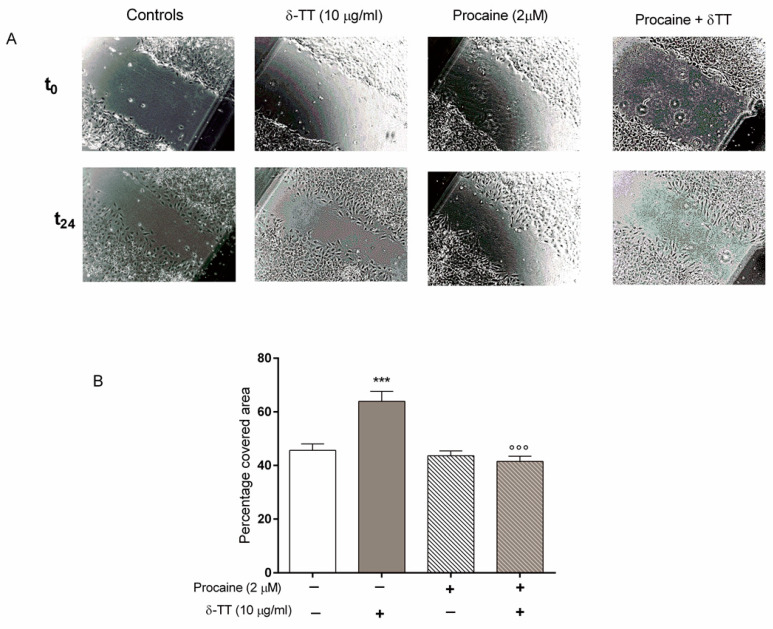
Procaine, a Wnt/β-catenin signalling pathway inhibitor, removed the wound healing effects of δ-TT (10 µg/mL) in MC3T3-E1 cells. Cells were incubated with procaine (2 μM) 30 min before δ-TT. The Wound healing assay was performed as detailed in Figure 2. (**A**) Wound healing assay of untreated and treated MC3T3-E1 cells; (**B**) Quantification of wound closure. Data are presented as the mean ± SEM of eight replicates. *** *p* < 0.001 vs. controls; °°° *p* < 0.001 vs. δ-TT.

The involvement of β-catenin in the enhancing action of δ-TT on MC3T3-E1 cell migration was confirmed by the results obtained with procaine, an inhibitor of the Wnt/β-catenin signalling pathway [20,21]. As shown in Figure 7, pretreatment with procaine (2 μM) per se did not modify the MC3T3-E1 cell ability to cover the wound, but completely prevented the positive action of δ-TT on MC3T3-E1 cell migration.

**Figure 8:**
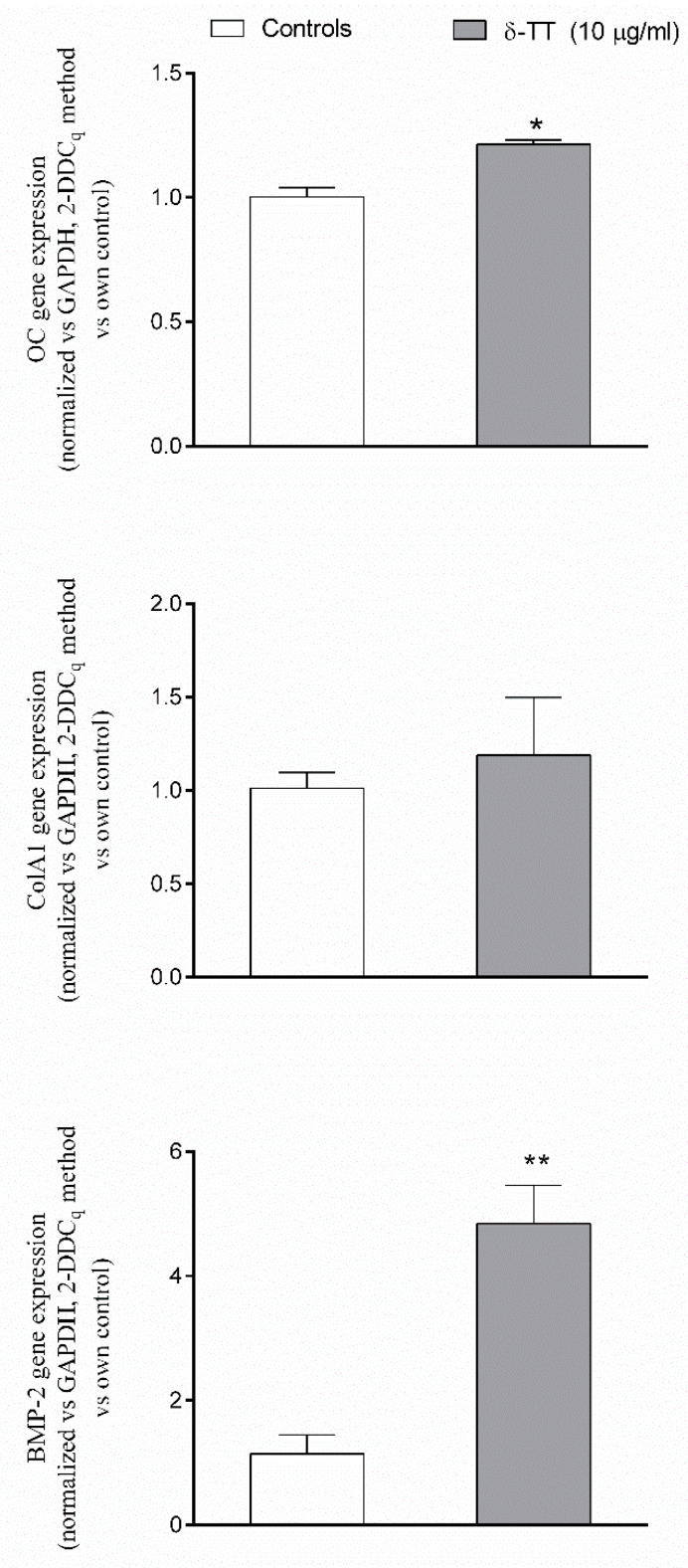
Effects of δ-TT (10 μg/mL) on OC, ColA1 and BMP-2 expression measured by real-time PCR. mRNAs were collected 24 h after treatment with δ-TT. 10 ng of cDNA from each sample were amplified using the SYBR^®^ Green Chemistry and a specific set of primers for OC, ColA1, and BMP-2. Each sample was evaluated in triplicate. Data were normalized for GAPDH and expressed as mean ± SEM of the relative amounts of gene/GAPDH of each sample vs. the mean control value Five replicates were performed for each experimental point and the experiments were repeated twice. * *p* < 0.05; ** *p* < 0.01 vs. controls.

**Figure 9:**
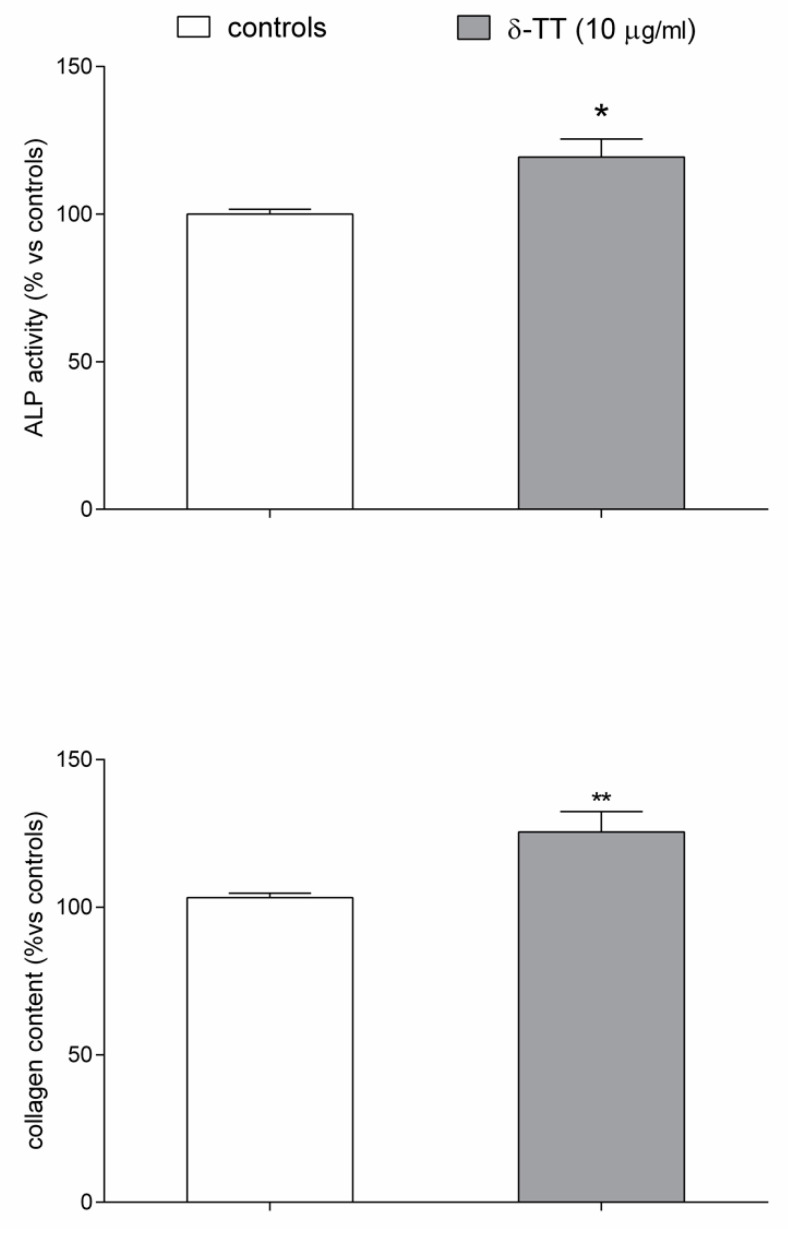
Effects of δ-TT (10 μg/mL) on MC3T3-E1 differentiation. ALP activity and collagen content were evaluated in differentiated cells 48 h after treatment. Data are expressed as the percentage relative to control and are the mean ± SEM of six replications. * *p* < 0.05, ** *p* < 0.01 vs. controls.

Finally, we examined the ability of δ-TT to modify the expression of several β-catenin target genes, involved both in cellular differentiation and migration, like osteocalcin (OC), collagen type 1 alpha 1 (ColA1) and bone morphogenetic protein-2 (BMP-2) using qPCR assay. δ-TT treatment induced a trend throughout an increase in the expression of OC, ColA1 and BMP-2, reaching a statistical significance, versus the untreated control cells, for OC and mainly for BMP-2 (Figure 8). The ability of δ-TT to stimulate MC3T3-E1 cell differentiation was examined by measuring ALP activity and collagen levels. As shown in Figure 9, we detected a significant increase in both markers of osteoblast differentiation in MC3T3-E1 treated cells in comparison with control-untreated cells. 

### 2.5. Epigenetic Mechanism: Effect of δ-TT on Histone Acetylation Levels

**Figure 10:**
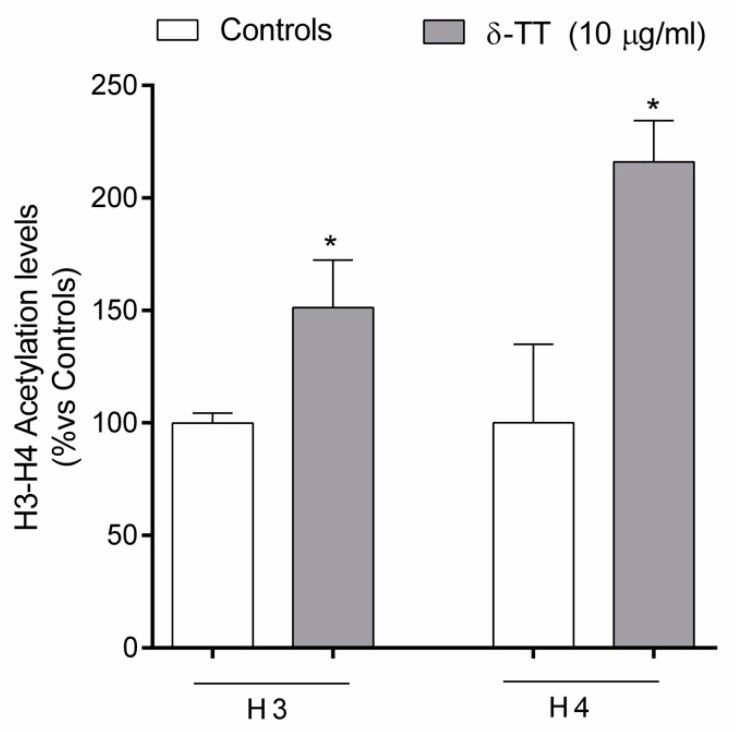
δ-TT increases global H3 and H4 acetylation levels in MC3T3-E1 cells. Cells were treated with δ-TT (10 µg/mL) for 24 h and the histones were collected. The acetylation of H3 and H4 was measured by a colorimetric assay. Absorbance was read at 450 nm. The data are presented as the mean ± SEM of three independent experiments. * *p* < 0.05 vs. controls.

Since, as cited above, bone cell activities may be regulated by the bone epigenome, in the last series of experiments, we studied the possible epigenetic mechanisms by which δ-TT exerts its effects on MC3T3-E1 cells. As shown in Figure 10, MC3T3-E1 cells treated for 24 h with δ-TT showed a significant increase in the amount of the histones H3 and H4 acetylation levels, important epigenetic marks of gene activation. 

## 3. Discussion

In the present study, we provide the first evidence that purified δ-TT, extracted from commercial palm oil (*Elaeis guineensis*) fraction, increased migration capacity of both MC3T3-E1 and BMSC cells in physiological conditions. We have also reported that the ability of δ-TT to promote MC3T3-E1 cell migration is dependent on Akt phosphorylation and involves Wnt/β-catenin signalling pathway activation. β-catenin signalling activated in response to δ-TT, results in the increased expression of specific target genes involved in osteoblast differentiation and migration such as OC, Colla1 and BMP2. Our results showing that the levels of H3 and H4 acetylation are significantly increased by δ-TT, suggest a possible involvement of epigenetic modifications in the positive effect of δ-TT on MC3T3-E1 cells activity.

Recently, TTs have been found to exert bone protective effects in several experimental models of osteopenia [22]. Furthermore, clinical studies have shown that dietary supplementation of TTs extracted from annatto seeds (consisting of 90% δ-TT and 10% γ-TT) exerts a beneficial action on bone turnover in postmenopausal women. AnnattoTT reduced osteoclast number and bone resorption and increased bone formation resulting in a net gain of bone [13]. 

Bone formation involves consecutive events of osteoblast migration, differentiation, and mineralization [23]. Emerging evidence suggests that an appropriate amount of osteoblast migration to the sites resorbed by osteoclast is fundamental for the maintenance of skeletal health [24]. The effects of TTs on osteoblast differentiation and mineralization have been previously studied. However, it remains to be clarified whether δ-TT, could be able to stimulate osteoblast migration.

In the present study, we have shown for the first time that δ-TT increased osteoblast migration and promoted scratch wound closure. We used MC3T3-E1 osteoblast-like cells considered a suitable model for studying osteogenic development in vitro. These cells are characterized by distinct proliferative and differentiation stages, thereby reproducing a temporal program similar to osteoblast differentiation as occurs during in vivo formation [25]. We have also examined the effects of δ-TT on the MC3T3-E1 cell cycle. We found that δ-TT inhibits MC3T3-E1 cell proliferation through inhibition of the transition between G0/G1 and S phase, maintaining cells in a quiescence phase. The associated events in the G0/G1 phase for a cell determine its fate with regards to proliferation and differentiation. Restriction point passage, which promotes cell cycle progression and S phase entry, is essential to drive cell proliferation, whereas cells that don’t pass the restriction point may return to G0/G1 phase in quiescence or may differentiate [26]. It has been reported that osteoblasts express Runt-related transcription factor 2 (Runx2) in the G0/G1 phase. Runx2 is important for osteoblast differentiation [27,28]. Indeed, we found that δ-TT stimulates MC3T3-E1 cell differentiation by increasing not only the expression of both OC and ColA1, but also the ALP activity and collagen levels, considered specific differentiation markers.

It is well known that BMSC cells play a critical role in bone repair after fractures [29].

BMSC cells, in fact, are capable to mobilize from bone marrow and specifically migrate to the fracture site where they differentiate into osteogenic cells able to restore both the callus volume and the biomechanical properties of the bone [30]. Interestingly, we found that δ-TT enhanced human primary BMSC migration, suggesting the potential utility of the local application of this compound to the damaged tissue to increase bone regeneration and bone repair.

As far as the signalling pathways involved in the stimulatory action of δ-TT on MC3T3-E1 cell motility, we first examined the Wnt/β-catenin signalling pathway, which exerts a regulatory role not only in cell proliferation but also in cell-cell adhesion and cell migration [31,32].

At the heart of the canonical Wnt signalling is the transcriptional regulator β-catenin which, in the absence of Wnt ligands, is targeted for proteasomal degradation. Wnt/LRP 6 signalling inhibits GSK3β which allows newly synthesized β-catenin to accumulate in the cytoplasm and translocate to the nucleus where it binds members of T cell Factor/lymphocyte enhancer factor family to initiate transcription of target genes. 

Even if the lack of measurements of the intranuclear translocation of β-catenin represents a limitation of our study, we suggest that the canonical Wnt signalling pathway is involved in the effect of δ-TT on MC3T3-E1 cell migration since δ-TT treatment increased β-catenin transcriptional activity as detected by luciferase assay and pretreatment with procaine, a local anaesthetic drug, previously reported to inhibit the Wnt/β-catenin pathway [20,21], prevents the increase of the wound healing activity induced by δ-TT. Our assumption is in line with previous in vivo studies, showing that oral annatto TT supplementation prevented osteopenia induced by metabolic syndrome by reducing SOST and DKK1 levels, considered antagonist of the Wnt signalling pathway [33].

PI3K/Akt signalling pathway is very critical for cell growth and migration. Akt phosphorylation is involved in the regulation of several signalling pathways and transcriptional networks controlling osteoblast development and activity such as the canonical Wnt signalling pathway and BMP signalling [34,35,36,37]. When we studied the involvement of PI3K/Akt pathway in the wound healing activity of δ-TT, we found that δ-TT increased the phosphorylated (activated) form of AKT and that pretreatment with LY294002, a PI3K-specific inhibitor, reversed the wound healing action of δ-TT on MC3T3-E1 cells. These results are in line with previous data showing that PI3K/Akt pathway is involved in the δ-TT-protective action against oxidative stress on MC3t3-E1 cells [16].

The stimulatory action of δ-TT on MC3T3-E1 cell migration could involve, at least in part, the increased expression of BMP-2, which by increasing the actin cytoskeleton rearrangement, facilitates cell motility [38]. Interestingly, it has been reported that PI3K signalling pathway is involved in BMP-2-induced cellular migration [39] and we have found that the effects of δ-TT on cytoskeleton rearrangement are similar to those induced by BMP-2 treatment [39].

We are aware of another limitation of our study, since we have determined only the expression of BMP-2 and its expression was not validated by protein expression. However, our assumption is in agreement with previous reports showing a positive influence of annatto TTs on BMP-2 expression detected not only in MC3T3-E1 cells but also in bone tissue samples from ovariectomized rats [40].

Recent evidence indicates that, among the mechanisms by which naturally occurring compounds, such as resveratrol and anthocyanin, impact bone cell activities, the bone epigenome exerts a fundamental role [19]. The epigenetic regulation of osteoblast activity refers to the mechanisms that impact gene expressions such as post-translational histone modifications, miRNA-mediated post-transcriptional regulation, and DNA methylation [41,42]. In the present study, we examined the possible epigenetic mechanisms by which δTT exerts its positive effects on MC3T3-E1 cells. Our data have shown that the positive action of δ-TT on osteoblast activity could be due, at least in part, to an increase in histone acetylation. Acetylation neutralizes the positive charge of histones and thereby prevents the compaction of chromatin. Interestingly, the negative impact of histone deacetylation on osteogenesis has been previously reported [43,44]. Further epigenetic studies will be focused to investigate the interplay between H3/H4 acetylation levels induced by δTT and β-catenin target genes.

## 4. Methods

### 4.1. δ-TT Purification

δ-TT was kindly provided by Giangiacomo Beretta (Department of Environmental Science and Policy, University of Milan, Milan, Italy). δ-TT was extracted by liquid chromatography using LC-940 Liquid Chromatography instrument (Varian, Leinì, Italy) from the fraction rich in tocotrienols/tocopherols contained in the commercial oil of Elaeis guineensis, (Gold Tri E 70% w/w, Golden Hope Bioganic, Selangor, Malaysia), as previously described [45]. A purity for δ-TT of at least >95% was achieved. δ-TT was divided into aliquots (50 mg/mL) and stored at −20 °C.

δ-TT was supplied to MC3T3-E1 or human bone marrow mesenchymal stem cells (BMSC) at the concentration of 10 μg/mL, corresponding to 25 μM (see [16]).

### 4.2. Cell Culture

Murine osteoblastic cell line from ATCC (cat. Num. CRL-2593), MC3T3-E1, were seeded in High Glucose DMEM, (Euroclone, Pero, Italy), in the presence of 10% FBS (Sigma-Aldrich Chemical, Milano, Italy), 2% L-glutamine, 100 µg/mL streptomycin and 100 U/mL penicillin at 37 °C in 5% CO_2_ atmosphere as previously reported [46]. Cell culture medium was replaced twice a week and MC3T3-E1 cells were trypsinized weekly. Primary BMSC isolated and cultured as previously described [47], were kindly provided by Anna Teresa Brini (Department of Biomedical, Surgical and dental Sciences, Università degli Studi di Milano). Briefly, BMSC were seeded in High Glucose DMEM, (Euroclone, Pero, Italy), in the presence of 10% FBS (Sigma-Aldrich Chemical, Italy), 1% L-glutamine, 50 µg/mL streptomycin and 50 U/mL penicillin at 37 °C in 5% CO_2_ atmosphere. Cell culture medium was replaced three times a week, and BMCS were trypsinized weekly.

### 4.3. Flow Cytometry Analysis

For cell cycle analysis, MC3T3-E1 cells were re-suspended at a density of 0.5 × 10^6^ cells/mL in PBS containing 0.1% Nonidet, RNAase A (50 µL, stock 100 µg/mL), and propidium iodide (PI, 400 µL, stock 50 µg/mL). After a 30-min incubation in the dark, cells were analyzed using a FACScalibur flow cytometer (Becton Dickson Biosciences, Milano, Italy) operated by Cell Quest software. Forward-scatter height (FSC-H) and side-scatter height (SSC-H) were acquired to study cell size and complexity.

### 4.4. Scratch Wound Healing Assay

A sterile pipette tip was used to generate a linear scratch on confluent MC3T3-E1 cells. After the remotion of cellular debris by washing with PBS, the cells were incubated in DMEM 1% FBS alone or at the presence of various treatments for 24 h. Photographs were taken immediately after the scratch was performed (t0) and at various times after treatment (t18 and t24) using an Olympus U-CMAD3 phase-contrast microscope equipped with a Zeiss Axiocam ICc1 camera at 4× magnification. The images were analyzed using ImageJ software (National Institute of Health, Bethesda, MD, USA) as previously reported [48]. For each experimental group, the percentage of the covered area was calculated by measuring the wound size at different times from treatment (t18 and t24 h) compared with the initial (t0) wound size considered as 100%.

### 4.5. Morphological Studies

MC3T3-E1 cells (5000 cells/well) were grown on 18-mm glass coverslips in 12-well plates. Cells were fixed with paraformaldehyde 3% and sucrose 2% for 10 min at 37 °C. After the fixation, cells were washed twice with PBS with calcium and magnesium and permeabilized with Triton X-100 0.2% in PBS for one minute at room temperature. The fixed cells were washed with PBS for 5 min and incubated with phalloidin-FITC 1/1000 (Sigma-Aldrich) for 20 min at 37 °C and with DAPI (1/10000) for 5 min. Images were obtained at 40× and 20× for cytoskeleton rearrangement with a Zeiss Axiovert microscope equipped for fluorescence analysis with the corresponding sets of filters to detect FITC and DAPI emission.

### 4.6. Boyden Chamber

We have investigated the δ-TT effects on BMSC migration. The analysis of cell migration was performed using a 48-wells Boyden chamber according to the manufacturer instructions (Neuroprobe, DBA, Agrate, Italy) as previously reported [49]. Briefly, cells were plated one week before at a density of 80,000 cells in 6 mm plate to avoid confluence. BMSC were pretreated for 24 h, with δ-TTs 10 µg/mL, and trypsinized. For migration experiments, 28 µL of DMEM with FBS 1% were placed in the lower compartment of the chamber, while the open-bottom wells of the upper compartment were filled with cells (50 μL of cell suspension, 20,000 cells for each well) collected by trypsin and resuspended in DMEM w/o FBS (2 × 10^4^ cells/well). Each pair of wells are separated by a polyvinylpyrrolidone-free polycarbonate porous membrane (8 μm pores) precoated with gelatin (0.2 mg/mL in PBS) the day before and maintained at 4 °C until use. After migration (overnight 37 °C), cells, adherent to the underside of the membrane were fixed by methanol and stained according to the Diff-Quik kit (Eurofins, Panlabs, Inc, St Charles, MO, USA). For quantitative analysis, cells were observed and counted using a 10× or 20× objective on an optical microscope. Three random objective fields of stained cells were counted for each well, and the mean number of migrating cells was calculated. Data are expressed as percentage versus control treated cells.

### 4.7. Transcriptional Activity Analysis

Transfections were performed by using Lipofectamine 2000 (Life Technologies, Monza, Italy) in 96-well plates according to the manufacturer’s protocol (see [50]). The following constructs commercially available (Promega, Milano, Italy) were transfected: pGL4.54 (luc2TK) used to normalize transfection efficiency (control reporter) and pNL (NLucP/TCF-LEF-RE) (experimental reporter) to analyze beta-catenin transcriptional activity. Transcriptional activity was measured using the Nano-Glo Dual-Luciferase Reporter assay system (Promega) according to the manufacturer’s protocols. Briefly, transfected MC3T3-E1 cells were cultured in 96-well plates and treated for 48 h with δ-TT (10 μg/mL) and LiCl (20 mM) as a positive control (not reported in the figure) for beta-catenin transcriptional activity. Each experiment was repeated three times.

### 4.8. Quantitative PCR Analysis

We have investigated the δ-TT effects on the β-catenin target genes expression (osteocalcin, OC, bone morphogenetic protein-2, BMP-2 and collagen type 1, alpha 1, ColA1). MC3T3-E1 cells (1.5 × 10^5^ cells/well) were plated in 6-well plates and treated with δ-TT, as previously described. Adherent cells were harvested, and total RNA was extracted using Purezol according to the manufacturer’s instructions (Biorad, Segrate, Italy). RNA pellet concentrations were assessed spectrophotometrically using microcuvette G1.0 in Eppendorf Biopohotometer. Specific sets of primers for OC (F: 5′- AGGAGGGCAATAAGGTAGT-3′ R: 5′- CATAGATGCGTTTGTAGGC-3), BMP -2 (F:5′-GGGACCCGCTGTCTTCTAGT-3′ R: 5′-TCAACTCAAATTCGCTGAGGAC-3), ColA1 (F:5′-GCATGGCCAAGAAGACATCC-3′ R:5′-CCTCGGGTTTCCACGTCTC-3′) and glyceraldehyde-3-phosphate dehydrogenase (GAPDH) (F:5′-CATCCCAGAGCTGAACG-3′ R:5′- CTGGTCCTCAGTGTAGCC-3′) were designed according [51,52] and synthesized by Eurofins Genomics (Vimodrone, Italy). Quantitative PCR was performed in QuantStudio Fast 5 (Thermofisher Scientific, Rodano, Italy) using 20 μL of total volume for each well. The efficiency of each set of primers was evaluated in preliminary experiments and it was found close to 100% for target and the housekeeping gene GAPDH. Total RNA (1500 ng) was retrotranscribed using the IScript Supermix kit (Bio-Rad), according to the manufacturer’s protocol. The amplification was carried out on 50 ng of total cDNA using SYBR chemistry (LUNA Universal QPCR Master Mix, NEB Science, Euroclone) according to the manufacturer’s protocol. Real-time PCR was run according to the following protocol: an initial step of 60 s at 95 °C followed by 40 cycles of 15 s at 95 °C and 30 s at 60 °C. A dissociation stage with a melt curve analysis was also performed. Five replicates were performed for each experimental point and experiments were repeated twice. Gene expression was quantified using the comparative threshold cycle (DDCt) method considering that the targets and the reference genes have the same amplification efficiency (near to 100%) verified as first in a standard curve experiment, as previously described [53].

### 4.9. Alkaline Phosphatase (ALP) Activity and Collagen Content

To study MC3T3-E1 cell differentiation, cells were seeded at the density of 1 × 10^4^ in 48 well-plates and treated at 90% confluence with culture medium containing 50 µg/mL ascorbic acid and 10 mM β-glycerophosphate to initiate osteoblast differentiation. After 3 days, the cells were cultured with a medium containing δ-TT (10 μg/mL) for 48 h and ALP activity or collagen content were measured.

ALP activity and protein concentration were detected in cells lysates (0.2% Triton-X-100) with a commercially available ALP activity assay kit (Sigma-Aldrich) and BCA-protein assay kit (Thermo-Fisher Scientific) respectively. ALP activity was measured as nmol/min/mg prot and results were expressed as percentage of controls. 

Collagen content was quantified by Sirius Red-based colorimetric assay [54] as previously described [53]. The results are expressed as percentage of control absorbance.

### 4.10. Western Blotting Analysis

Cells were collected in protein buffer with protease and phosphatase inhibitors (NaCl 150 mM, Tris-Hcl 50 mM, 1% Triton × 100). Lysates were centrifuged at 10,000 g for 10 min at 4 °C and protein concentration determined with the BCA assay. The extracts were electrophoresed on 15% SDS/PAGE. All protein gels were electrotransferred to 0.2-μpore nitrocellulose membranes by a wet procedure (1 h for 350 constant mA and 30 min 400 constant mA, room temperature). After transfer, membranes were systematically stained with Ponceau S dye to verify the correct loading and transfer quality of proteins. Membranes were blocked and then probed overnight at 4 °C with the primary specific antibodies and processed, as described by the manufacturer, with fluorescent secondary antibodies (WesternDot™ 625 from Western Blot Kits, Thermo-Fisher Scientific).

Fluorescent signals were quantified using a CHEMIDOC system equipped with UV filters (Bio-Rad Italia, Milano, Italy). The primary antibodies employed for western were as follows: anti AKT Pan (rabbit anti- AKT 1:1000; catalog 9272 Cell Signalling Technology, Euroclone) and anti-phosphorylated AKT (Ser473) (rabbit anti- AKT 1:1000; catalog 9271 Cell Signalling Technology, Euroclone) as previously reported [55]. Furthermore, to detect the correct loading of the proteins, we have also detected the GAPDH lane (Abcam Loading control, catalog Ab8245, 1:1000, Milano, Italy).

### 4.11. H3 and H4 Global Acetylation Levels

Histones were extracted from MC3T3-E1 using the Histone Extraction Kit (Abcam; #113476) according to the manufacturer’s protocol. Briefly, MC3T3-E1 cells (1.0 × 10^5^ cells/well) were plated in 6-well plates and treated with dTTs as previously described. Adherent cells were harvested and put into 100 μL of 1× pre-lysis buffer, homogenized, and centrifuged to obtain a pellet. Pellets were resuspended in 200 μL of lysis buffer, mixed, and put on ice for 30 min. The samples were centrifuged to obtain a supernatant that contained the acid extracted histones, which were then transferred into a pH neutralizing buffer solution containing dithiothreitol (DTT). Histone protein concentrations were measured using the BCA Protein Assay Kit (Pierce BCA Protein Assay Kit # 23227, Thermofisher Scientific). Absorbance was measured at 750 nm on a microplate spectrophotometer reader (Victor™, PerkinElmer, Milano, Italy), and concentrations were calculated from a standard curve. 

The histone acetylation level detection was performed using the EpiSeeker Histone H3 (ab115124) and H4 Total Acetylation Detection Colorimetric Kits (ab115125), purchased from Abcam. Specific buffers ESC1 and ESC2 were added into each well, together with the 5 μg of the histone extract into the sample wells. The diluted standards were added, and strips were incubated at room temperature for one hour. After washing and aspiration, the diluted buffer was added to each well and incubated at room temperature for 60 min. The wells were aspirated, and color reagent and stop solution were added. The obtained yellow color was read on a microplate reader at 450 nm within 2–15 min.

### 4.12. Statistical Analysis

All statistical analyses were performed by GraphPad Prism8 (GraphPad Software, San Diego, CA, USA). The results are expressed as the mean ± SEM of at least 3 independent experiments (3 replicates for each experiment). Differences between groups were evaluated by one-way ANOVA followed by Bonferroni post-hoc test or unpaired Student *t*-test, when appropriate. A *p*-value of less than 0.05 was considered significant.

## 5. Conclusions

In summary, we have shown for the first time, that δ-TT increases the motility of both MC3T3-E1osteoblast-like cells and primary human BMSC in physiological conditions. Moreover, we found that activation of both PI3K/Akt and Wnt/β-catenin pathways induced by δ-TT play a critical role in the wound healing action of δ-TT on MC3T3-E1 cells. The ability of δ-TT to enhance the recruitment of BMSC, and to promote MC3T3-E1 differentiation and migratory behavior, indicates that δ-TT could be considered as a promising natural compound to facilitate fracture healing.
